# Correlation of anti-tumour drug resistance with epigenetic regulation

**DOI:** 10.1038/s41416-020-01183-y

**Published:** 2020-12-03

**Authors:** Takuma Hayashi, Ikuo Konishi

**Affiliations:** 1 National Hospital Organization, Kyoto Medical Center, Kyoto, Japan; 2 START-program, Japan Science and Technology Agency (JST), Tokyo, Japan; 3 Graduate School of Medicine, Kyoto University, Kyoto, Japan

**Keywords:** Cancer therapeutic resistance, Drug development

## Abstract

During treatment, the development of drug-resistant tumours remains an important challenge in cancer treatment. Epigenetic changes have been reported as one of the mechanisms for anti-tumour drug resistance. In clinical practice, a combination of epigenetic-related drugs can be considered as a future selection of cancer therapeutic drugs.

## Main

Pharmacotherapy for cancer has undergone great changes with the advent of molecular-targeted therapeutic agents specific for cancer. Molecularly targeted drugs are extremely effective against cancers with target factors such as driver oncogenes that strongly induce canceration. During treatment, development of tumour resistance remains a significant challenge in cancer therapy. Thus, it is imperative to elucidate the mechanism of anti-tumour drug resistance to develop therapeutic approaches that overcome or prevent resistance.^1^ Because of significant progress in the advent of molecularly targeted drugs for certain types of cancer, such as lung, colorectal and ovarian cancer, many researchers are pursuing efforts to identify and characterise not only the mechanisms of resistance, but also strategies to overcome it.

Colorectal cancer cells originally have natural resistance, also known as pre-existent or intrinsic resistance, which impairs the effectiveness of anti-tumour drugs. In the treatment of conditions, such as leukaemia, there are many cases in which early effectiveness of an anti-tumour drug becomes ineffective later in treatment, which is known as acquisition resistance. Molecular biology-based studies investigating factors involved in anti-tumour drug sensitivity and drug resistance of cancer and normal cells have contributed to predicting the effects of anti-tumour drugs. The development of drug therapy targeting factors involved in drug resistance is expected to contribute to a better understanding of the therapeutic effects of cancer chemotherapy. Cancer cells acquire anti-tumour drug resistance through one of three mechanisms: (a) gene expression likelihood, (b) gene expression decrease or (c) gene mutation.

Cancer cells are characterised by unregulated growth, damage to normal cells and growth via distant metastasis. In tumour cells such as myeloma cells, there is either abnormal or impaired proteasome function, which is an enzyme complex that plays an important role in the cell cycle. Proteins that are no longer needed by the cell are marked through ubiquitination and degraded by proteasomes. Drugs that induce programmed cell death (apoptosis) in cancer act through proteasome inhibition, thereby preventing protein degradation and resulting in the accumulation of unwanted, abnormal or misfolded proteins. For instance, the proteasome inhibitor (PI) bortezomib prevents activation of the transcription factor nuclear factor-κB and, consequently, degradation of tumour protein p53 (TP53).^2^ As a result, bortezomib induces cell apoptosis, resulting in its anti-tumour effect. Carfilzomib binds to the β5 subunit of 20S proteasome,^3^ and as a result, inhibits proteasome activity by preventing chymotrypsin-like activity. Furthermore, carfilzomib also exerts a cytotoxic effect on cells from cancer cell lines that are resistant to bortezomib. PIs are a class of molecular target drugs that exert anti-tumour effects by inhibiting the signal transduction pathways of specific molecules involved in cancer cell growth.

**Fig. 1:**
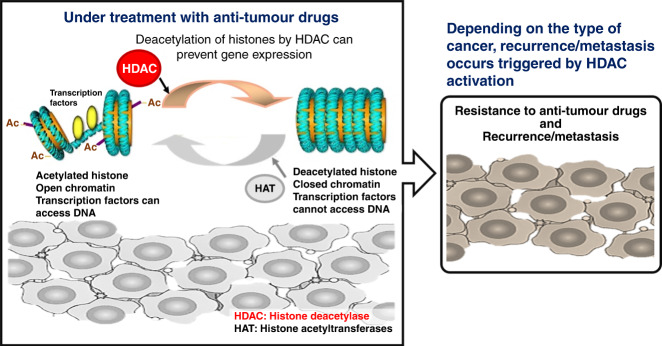
Although a detailed mechanism is not fully understood, histone deacetylase (HDAC) expression may be induced during clinical treatment with anti-tumour drugs, leading to inhibition of several genes as a result of histone deacetylation. Anti-oncogenic factors, including tumour protein p53 (TP53) and interferon regulating factor 1 (IRF1), are significantly downregulated during anti-tumour drug treatment, thereby leading to anti-tumour drug resistance of malignant tumours and cancers. Depending on the type of cancer or malignant tumour, recurrence/metastasis is triggered by HDAC activation. However, in cases of multiple myeloma, acquisition of anti-tumour drug resistance is not observed because of combination therapy using anti-tumour drugs and HDAC inhibitors. Furthermore, the acquisition of drug resistance in malignant tumours or cancers is induced by somatic mutations in key factors; however, development of drug resistance can also occur because of epigenetic changes of tumour suppressor genes caused by aberrant expression of epigenetic regulators such as HDAC activation. Therefore, in future clinical medicine, combination therapy with anti-tumour drugs and inhibitors against epigenetic factors such as HDAC inhibitors may be an effective chemotherapeutic approach for malignant tumours or cancer. Ac acetylated histone.

Resistance to PIs is a major obstacle to the successful treatment of multiple myeloma (MM).^2,3^ During PI-based treatments, MM cells enter a slow cycling and reversible drug-tolerant state. This reversible phenotypic transition is associated with epigenetic plasticity, involving the development of tolerance rather than persistence in patients with relapsed MM (Fig. 1). Ge et al.^4^ examined the effects of PI-based intermittent therapy or treatment in combination with histone deacetylase (HDAC) inhibitors on drug-tolerant MM cells, and demonstrated that the combination of HDAC inhibitors and high-dosage intermittent therapies, as opposed to sustained PI monotherapy, can be more effective in treating MM by preventing the emergence of PI-tolerant cells (Fig. 1). In other words, the therapeutic basis is the reversal of dysregulated epigenetic regulators in patients with PI-treated MM. Previous studies have confirmed that HDAC inhibitors possess anti-tumour effects on various cancer types.^5^ Ge et al. have shown that the combination of epigenetic factor inhibitors, such as HDAC inhibitors may be effective against drug resistance observed in the process of treating cancer and malignant tumours with anti-tumour drugs.

Advanced and recurrent ovarian cancer changes into refractory cancer by acquiring resistance to chemotherapy centred on platinum drugs. Horiuchi et al.^6^ reported that expression of S100 calcium-binding protein A4 (S100A4) was correlated with invasiveness of ovarian carcinoma cells in vitro and in vivo, and that increased expression of S100A4 was associated with hypomethylation of CpG sites in the first intron of *S100A4* in ovarian carcinoma with cisplatin resistance.^7^ These results demonstrate that epigenetic alteration plays an important role in the metastatic phenotype and acquired transformation of ovarian cancer cells.

In the treatment of cancer, drug resistance to anti-tumour drugs that either do not work or lose effectiveness during treatment is of major concern for patients and healthcare professionals. Previous research on anti-tumour drug resistance was conducted to elucidate the molecules involved in resistance development. An understanding of the underlying resistance mechanisms of anti-tumour drugs is critically important to further improve treatment outcomes of advanced/recurrent cancers. These studies have discovered that changes in the expression of factors involved in tumorigenesis and metastasis because of epigenesis are causally related to drug resistance and tumour malignancy. In the future of clinical medicine, a combination of epigenetic-related drugs such as HDAC inhibitors and clinically recommended anti-tumour drugs may be recommended to avoid anti-tumour drug resistance.

## Data Availability

Not applicable.

## References

[1] Huang T, Song C, Zheng L, Xia L, Li Y, Zhou Y (2019). The roles of extracellular vesicles in gastric cancer development, microenvironment, anti-cancer drug resistance, and therapy. Mol. Cancer.

[2] Manasanch EE, Orlowski RZ (2017). Proteasome inhibitors in cancer therapy. Nat. Rev. Clin. Oncol..

[3] Barrio S, Stühmer T, Da-Viá M, Barrio-Garcia C, Lehners N, Besse A (2019). Spectrum and functional validation of PSMB5 mutations in multiple myeloma. Leukemia.

[4] Ge, M., Qiao, Z., Kong, Y., Liang, H., Sun, Y., Lu, H. et al. Modulating proteasome inhibitor tolerance in multiple myeloma: an alternative strategy to reverse inevitable resistance. *Br. J. Cancer*10.1038/s41416-020-01191-y (2020). 10.1038/s41416-020-01191-y PMC7884794 33250513

[5] Hayashi A, Horiuchi A, Kikuchi N, Hayashi T, Fuseya C, Suzuki A (2010). Type-specific roles of histone deacetylase (HDAC) overexpression in ovarian carcinoma: HDAC1 enhances cell proliferation and HDAC3 stimulates cell migration with downregulation of E-cadherin. Int. J. Cancer.

[6] Horiuchi A, Hayashi T, Kikuchi N, Hayashi A, Fuseya C, Shiozawa T (2012). Hypoxia upregulates ovarian cancer invasiveness via the binding of HIF-1α to a hypoxia-induced, methylation-free hypoxia response element of S100A4 gene. Int. J. Cancer.

[7] Hayashi T, Konishi I (2017). Epigenetic changes of pro-oncogenic genes during ovarian cancer progression. Women Heal. Care.

